# Supplementary material for: TRAIL Triggers CRAC-Dependent Calcium Influx and Apoptosis through the Recruitment of Autophagy Proteins to Death-Inducing Signaling Complex
Source: Cells. 2021 Dec 25;11(1):57. doi: 10.3390/cells11010057 (PMC8750441; doi:10.3390/cells11010057)
Supplement: Supplementary file 1 [file cells-11-00057-s001.zip › cells-1486076-supplementary.pdf]

cells

MDPI

*Article*

# TRAIL Triggers CRAC-Dependent Calcium Influx and Apoptosis through the Recruitment of Autophagy Proteins to Death-Inducing Signaling Complex

**Kelly Airiau [1,†], Pierre Vacher [1,†], Olivier Micheau [2,3], Valerie Prouzet-Mauleon [1], Guido Kroemer [4,5,6], Mohammad Amin Moosavi [7,*] and Mojgan Djavaheri-Mergny [4,5,*]**

[1] Institut Bergonié, INSERM U1218, University of Bordeaux, 33000, Bordeaux, France; kelly.airiau@gmail.com (K.A.); pierre.vacher@inserm.fr (P.V.); valerie.prouzet-mauleon@u-bordeaux.fr (V.P.-M.)
[2] Lipides, Nutrition Cancer, INSERM, UMR1231, 21079 Dijon, France; Olivier.micheau@inserm.fr
[3] UFR Science de Santé, Université de Bourgogne, Franche-Comté, 21079 Dijon, France; [4] Centre de Recherche des Cordeliers, INSERM UMRS 1138, Sorbonne Université, Université de Paris, Equipe 11 labellisée par la Ligue contre le Cancer, 75006, Paris, France; kroemer@orange.fr
[5] Metabolomics and Cell Biology Platforms, Institut Gustave Roussy, 94805 Villejuif, France;
[6] Pôle de Biologie, Hôpital Européen Georges Pompidou, AP-HP, 75015 Paris, France
[7] Department of Molecular Medicine, National Institute of Genetic Engineering and Biotechnology, Tehran P.O. Box 14965/161, Iran
* Correspondence: a-moosavi@nigeb.ac.ir (M.A.M.); mojgan.mergny@inserm.fr (M.D.-M.)
† Co-first authors

# Supplementary Data

**Table S1.** Information on the numbers and type of The Cancer Genome Atlas (TCGA) tumors and adjacent (Adj) tumor samples as well as Genotype-Tissue Expression (GTEx) normal tissues. The data have been extracted from Gene Expression Profiling Interactive Analysis (GEPIA) (http://gepia.cancer-pku.cn/index.html).

| TCGA cancer type | Tumor Numbers | Adj Tumor Numbers | GTEx Normal Tissue Type | GTEx Sample Numbers |
|---|---|---|---|---|
| Adrenocortical carcinoma (ACC) | 77 | - | Adrenal Gland | 128 |
| Bladder Urothelial Carcinoma (BLCA) | 404 | 19 | Bladder | 9 |
| Breast invasive carcinoma (BRCA) | 1085 | 112 | Breast | 179 |
| Cervical squamous cell carcinoma and endocervical adenocarcinoma (CESC) | 306 | 3 | Cervix Uteri | 10 |
| Colon adenocarcinoma (COAD) | 275 | 41 | Colon | 308 |
| Lymphoid Neoplasm Diffuse Large B-cell Lymphoma (DLBC) | 47 | - | Blood | 337 |
| Esophageal carcinoma (ESCA) | 182 | 13 | Esophagus | 273 |
| Glioblastoma multiforme (GBM) | 163 | - | Brain | 207 |
| Head and Neck squamous cell carcinoma (HNSC) | 519 | 44 | - | - |
| Kidney Chromophobe (KICH) | 66 | 25 | Kidney | 28 |
| Kidney renal clear cell carcinoma (KIRC) | 523 | 72 | Kidney | 28 |
| Kidney renal papillary cell carcinoma (KIRP) | 286 | 32 | Kidney | 28 |
| Acute Myeloid Leukemia (LAML) | 173 | - | Bone Marrow | 70 |

| | | | | |
|---|---|---|---|---|
| Brain Lower Grade Glioma (LGG) | 518 | - | Brain | 207 |
| Liver hepatocellular carcinoma (LIHC) | 369 | 50 | Liver | 110 |
| Lung adenocarcinoma (LUAD) | 483 | 59 | Lung | 288 |
| Lung squamous cell carcinoma (LUSC) | 486 | 50 | Lung | 288 |
| Ovarian serous cystadenocarcinoma (OV) | 426 | - | Ovary | 88 |
| Pancreatic adenocarcinoma (PAAD) | 179 | 4 | Pancreas | 167 |
| Prostate adenocarcinoma (PRAD) | 492 | 52 | Prostate | 100 |
| Rectum adenocarcinoma (READ) | 92 | 10 | Colon | 308 |
| Skin Cutaneous Melanoma (SKCM) | 461 | 1 | Skin | 557 |
| Stomach adenocarcinoma (STAD) | 408 | 36 | Stomach | 175 |
| Testicular Germ Cell Tumors (TGCT) | 137 | - | Testis | 165 |
| Thyroid carcinoma (THCA) | 512 | 59 | Thyroid | 278 |
| Thymoma (THYM) | 118 | 2 | Blood | 337 |
| Uterine Corpus Endometrial Carcinoma (UCEC) | 174 | 13 | Uterus | 78 |
| Uterine Carcinosarcoma (UCS) | 57 | - | Uterus | 78 |

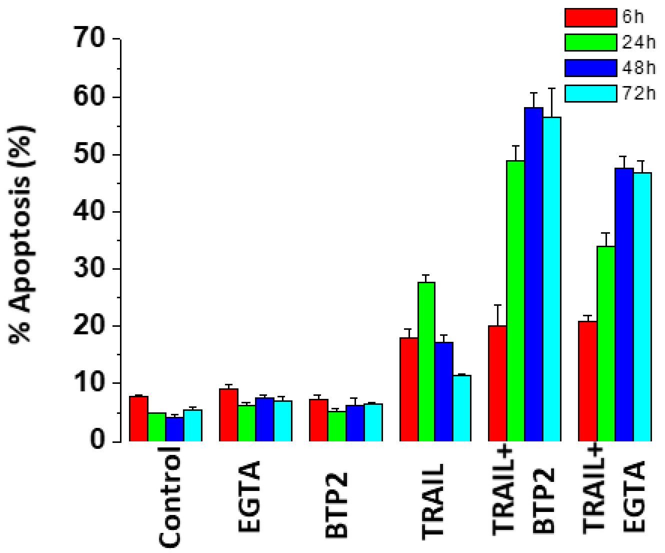


**Figure S1. Time-dependent effects of the calcium influx blockers BTP2 and EGTA on TRAIL induced apoptosis.** NB4 cells were treated with 250 ng/ml TRAIL in the presence or absence of ORAI1 channel inhibitor BTP2 (5 μM) or extracellular $Ca^{2+}$ chelator, 100μM EGTA. Apoptotic cell death was analyzed after 6 h to 72 h using TMRM staining by flow cytometry. Results are from three independent experiments and expressed as means ± S.D.

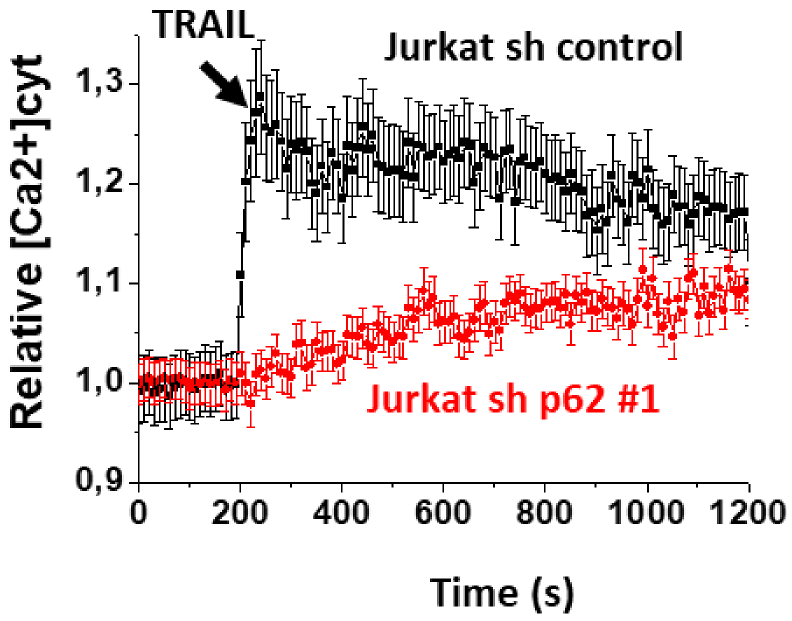


**Figure S2.** p62 deficiency inhibits calcium entry induced by TRAIL in Jurkat cells. Jurkat control (scramble shRNA) cells and Jurkat cells expressing sh p62 (clone#1) were treated with 250 ng/ml TRAIL and then calcium levels were measured after Fura-2AM.
